# Why and How Does Empowering Leadership Promote Proactive Work Behavior? An Examination with a Serial Mediation Model among Hotel Employees

**DOI:** 10.3390/ijerph18052386

**Published:** 2021-03-01

**Authors:** Chung-Jen Wang, I-Hsiu Yang

**Affiliations:** Department of Hotel and Restaurant Management, National Pingtung University of Science and Technology, 1, Shufu Road, Neipu, Pingtung 912301, Taiwan; wchungzen@gmail.com

**Keywords:** sustainable human resource management, human health, empowering leadership, job characteristics, job embeddedness, proactive work behavior

## Abstract

With the increasing competition in contemporary enterprise, sustainable human resource management is a powerful resource for workplace mental health. On the basis of job demands-recourses theory and conservation of resources theory, this study examined the relationship between empowering leadership and employees’ proactive work behavior. It also explored how job design inspires employees to be embedded in their work and to exhibit proactive work behavior. In addition, the research probed the mediating roles of job characteristics and job embeddedness in a serial mediation model within an integrated model. Data were collected from 461 employees of three- to five-star hotels through stratified random sampling. Results indicated that (1) empowering leadership has positive influences on job characteristics and proactive work behavior; (2) job characteristics have a positive influence on job embeddedness; (3) job embeddedness has a positive influence on proactive work behavior; (4) job characteristics mediate the effect of empowering leadership on proactive work behavior; (5) job embeddedness mediates the effect of empowering leadership on proactive work behavior; and (6) job characteristics and job embeddedness jointly mediate the effect of empowering leadership on proactive work behavior by bootstrapping analyses. Accordingly, this study suggests that promoting sustainable human resource management is needed for human health and organizational value at work, both of which enable empowering leadership to improve proactive work behavior via job characteristics and job embeddedness. The theoretical and managerial implications of empirical findings are also discussed.

## 1. Introduction

Scholars have argued about the urgent requirement to discover strategies for supporting people and promoting positive mental health in the workplace [1,2,3]. Workplace mental health is a condition of mind regarding how employees’ think and feel in everyday working life [4,5]. By addressing the importance of workplace mental health, organizations can engage employers in good practice for overcoming risks; improve employees’ productivity with stress reduction, job satisfaction, effectiveness, and work enhancement; and reduce absenteeism [6,7]. In this line, the ability of maintaining mental health has a beneficial effect on employees’ proactive behavior at work and can be regarded as a key component of work-related performance [8]. Furthermore, high-performance work practices, which are important strategies that link human resource management system, can ultimately benefit organizational operation [9,10,11,12]. Specifically, high-performance work practices motivate employees to leverage their knowledge, skills, and abilities and yield high performance [9] and thus can be considerably viewed as advanced competitive advantages in managerial practices [13]. Therefore, job design with high-performance work practices can lead to a highly motivated and empowered workforce [14], job embeddedness with low turnover intention [15], extra-role customer service [16], and high productivity [17,18,19]. As competition increases, if employees can take the initiative to help their organization discover and solve problems, the organization can effectively face the threats and challenges posed by the environment [20,21]. Furthermore, sustainable human resource management has garnered a great amount of attention, which can be used to decide the strength of a human resource management system and create an organizational value [19,22]. Karman [22] defined sustainable human resource management as three dimensions: (1) value for organization (including productivity, change management, flexibility, innovativeness, improved economic value, and profitability); (2) value for society (including family and community well-being, employment growth, firm image, and human health); and (3) value for employee (including good work-life, quality of life, engagement, employee well-being motivation, and employability). Accordingly, industry practitioners have labeled sustainable human resource management for offering a new approach to manage people, preserve productivity, and retain quality employees [23]. That is, sustainable human resource management regards employees as the most important resources of companies and emphasizes continuous investment in employee development. The benefit of sustainable human resource management is conducive to the improvement of employees’ working ability, including the stock of human capital for employees’ growth needs [24,25]. Accordingly, sustainable human resource management realizes the common development of human health and proactive behavior at work, even the long-term development of organizations.

According to job demands-recourses theory [26], job demands are stressors of physical, social or organizational factors at work, whereas job resources are support from abovementioned factors that help employees accomplish goals. Employees thus utilize job resources to defy job demands and make changes in their work to display proactive work behavior [27]. Meanwhile, conservation of resources theory describes the motivation that prompts employees to keep their current resources and chase new ones [28,29]. Therefore, resources are defined as anything that is based on individual perception, which can meet goals, and people intend to preserve such resources and prevent resource losses. [30]. An enriched and excellent job design of sustainable human resource management enables employees to acquire job-specific knowledge, skills, and abilities, which they will not want to lose [30]. This design can also find the meanings for high-performance work practices [31]. Furthermore, empowering leadership means a process of sharing power via a specific set of leader behaviors for their employees [32]. In this vein, scholars have argued that empowering leadership is the most relevant and effective in affecting service-oriented behaviors [33]. Specifically, it enhances the meaning and significance of work by offering employees the autonomy and flexibility to make decisions during service delivery. Employees can also switch flexibly between jobs during intradepartmental cooperation, which is indispensable in modern-day organizations [34]. Empowering leadership positively affects employees’ psychological empowerment and ultimately influences their work engagement [35]. Organizations must develop empowering leaders and promote empowering leadership behavior. Lin et al. [36] extended empowering leadership research on the basis of motivational and exchange-based models [37,38] to investigate the effects of empowering leadership on employees’ work outcomes in the contemporary industry. High-performance work practices are also important strategies for managers and human resource managers in the present-day industry to foster empowered and engage employees in a consistent way [39]. These employees are the ones embedded at work and may not show turnover intention [40]. That is, employees who perceive high-performance work practices at work exhibit high creative and extra-role performances [41]. To sum up, employee empowerment has wide applications in the modern-day industry, and the empowerment strategy can make employees take further initiative [34].

A leader can establish a work environment where his followers are supported, and by empowering them and encouraging their autonomy, followers can exert extra effort and responsibility at work [42]. Consequently, the support of leaders enhances the proactive motivation and behavior of followers at work [43]. According to the cognitive–motivational mechanisms of individual-level proactive behavior [44,45], situational factors (e.g., job design, leadership) and motivational processes are suggested as antecedents to proactive work behavior, which, in turn, has positive effects on individual, team, and organizational outcomes. This view is consistent with job design theory and job characteristics prompting psychological states to lead to personal and work outcomes [46]. Thus, job characteristics are important environment antecedents to proactive work behavior [44,47], and job autonomy provides employees with flexible work control because they have opportunities to make changes in their workplace [48]. Similarly, leaders must have the tendency to adopt an empowering leadership strategy to develop a close leader–subordinate relationship, which further effectively decreases the negative effects on employees [49]. These employees are given adequate space and discretion by their empowering-style leaders, causing them to demonstrate a willingness to work on tasks on their initiative [50]. In addition, job embeddedness represents the collection of factors that keep employees at their work [51]. Mitchell et al. [52] thus indicated the effect of employee voluntary turnover for organizations, and job embeddedness research has become necessary in the contemporary industry to understand employee retention, which keeps employees on their job instead of quitting [53]. Therefore, based on job demands-recourses theory and conservation of resources theory, the overall study objective is to formulate a broad research and development framework for: (1) the effects of empowering leadership on job characteristics and proactive work behavior; (2) the effect of job characteristics on job embeddedness; (3) the effect of job embeddedness on proactive work behavior; (4) the effect of empowering leadership on proactive work behavior via job characteristics; (5) the effect of empowering leadership on proactive work behavior via job embeddedness; and (6) the joint effect of empowering leadership on proactive work behavior via job characteristics and job embeddedness.

Furthermore, the present study fills previous research gaps in threefold. First, although the relationship between empowering leadership and working behavior has been tested [49], investigations that extend proactive behavior studies under an evidenced framework are lacking [44,45]. The results of the present study thus contribute to literature, which indicates that empowering leadership directly and indirectly influences proactive work behavior in a serial mediation way. Second, our study results propose that job characteristics and job embeddedness play significant positions in influencing proactive work behavior when leaders often attempt to empower employees. This research is one of the leading investigations that attempt to consider two concepts in an integrated model. Last, prior studies focused on job characteristics and job embeddedness as critical roles in organizational settings [54,55]. However, only few of them can provide comprehensive investigations of these two concepts simultaneously. Therefore, our study results provide a new perspective that the joint effect of job characteristics and job embeddedness can mediate the effect of empowering leadership on proactive work behavior.

In summary, this study expands prior studies in this field [3,22,45,56,57] and proposes an integrated model by combining job demands-recourses and conservation of resources theories for explaining and predicting employees’ motivation and attitude for workplace mental health. Sustainable human resource management is a survival strategy for industry practitioners to reach long-term successful businesses [19]. Therefore, this study adopts the concept of sustainable human resource management to retain employees and confirm the relationship between empowering leadership and employees’ proactive work behavior with a serial mediation model. Accordingly, this research supports that sustainable human resource management practices can be regarded as powerful resources to escalate competitiveness level, which eventually enables positive mental health in the workplace.

## 2. Conceptual Framework and Hypothesis Development

### 2.1. Relationship between Empowering Leadership and Proactive Work Behavior

Empowering leadership refers to a process of sharing power through a specific set of leader behaviors for employees [32]. It suggests that empowerment must include a motivational effect [58,59]. Accordingly, empowerment can be split into two approaches. The structural approach focuses on leaders’ empowering behavior. Leading by example, participative decision making, coaching, informing, and showing concern are identified as key dimensions of empowering leadership [60]. Another point to consider is psychological empowerment, which focuses on the extent to which employees feel empowered. Specifically, the concept of psychological empowerment is clearly distinguished from that of empowering leadership, which, in turn, enhances the feeling of psychological empowerment among followers [61]. Leaders’ empowering leadership behavior positively influences followers’ psychological empowerment and behavioral attitude, leading to creative behaviors and work outcomes at individual and team levels [56,62]. Furthermore, both empowerment strategies enhance subordinates’ self-efficacy [58,63], which strengthens individuals’ work belief (e.g., interests, goals, and behaviors) in the workplace [64]. Empowering leadership gives employees great autonomy from bureaucratic constraints, stimulates employees to participate in decision making, enhances the meaningfulness of work, and allows them to express confidence in high performance [32]. Thus, employees have the flexibility to accomplish the work and perform organizational citizenship behavior [65]. Workplace empowerment also promotes employees’ creative performance. When employees find the responsibility and authority to make decisions, they are motivated to generate and provide new ideas to perform quality service for customers and successfully solve their problems [55]. Empowering leadership is recently confirmed to foster proactive behavior in the modern-day industry [49]. Proactive work behavior refers to the initiative to change the internal environment or an individual [44]. In a narrow sense, employees intend to improve situations and attempt to create new opportunities in the current work surroundings. Compared with passive behavior, proactive employees are inclined to apply themselves to the goals of work instead of waiting for opportunities [20]. Proactive work behavior is focused on taking control of and bringing about change in the work environment [66]. Proactive employees exhibit a motivated behavior at work and can independently complete their tasks [67]. Accordingly, proactive work behavior becomes a highly profitable strategy for employees to manage their job performance and work experience [66,68].

Overall, the empowerment strategy makes employees take further initiative because they have the flexibility to accomplish their work [34], and empowering leadership plays a necessary role in predicting work performance and productivity [43,45]. Thus, examining how employees are motivated by empowering leaders to perform proactive work behavior and positive work outcomes is important. Although the relationship between empowering leadership and proactive work behavior has been examined [49], research that extends proactive behavior studies, which provide an evidenced integrated model, remains lacking [44,45]. Accordingly, the current study proposes a comprehensive integrated model by combining job demands-recourses and conservation of resources theories for explaining and predicting the motivation and attitude of employees. It broadens previous research and contributes to literature, which indicates that empowering leadership directly influences proactive work. Therefore, we propose the following:

**Hypothesis** **1** **(H1).** *Empowering leadership has a positive influence on employees’ proactive work behavior.*

### 2.2. Relationship between Empowering Leadership and Job Characteristics

Hackman and Lawler [69] proposed the concept of job characteristics that describes the external tasks, which can affect employees’ perceptions of work, thereby generating internal job satisfaction and enhancing work outcomes. Accordingly, Hackman and Oldham [46,70,71] proposed the most widely known and influential job characteristic model, which posits that work attributes can have motivational effects on employees. Enriched and enlarged jobs can be a crucial factor to prompt employees’ work motivation and improve their job satisfaction. Despite the focus on job enrichment, job characteristics theory remains fruitful in work design for the next decade [31]. The process of job characteristics is created through work design, which refers to the way that employees have to perform their tasks at work [72]. The present day industry is regarded as one of the most stressful industries [73]. According to the job demands-recourses model of work engagement, job resources, such as autonomy and feedback, cause work engagement, and engaged employees are expected to exhibit improved job performance [26]. The job demands-recourses model also indicates that long-term and high work demands with few resources can lead to exhaustion [74]. Thus, clarifying the causes of job burnout in the contemporary industry and preventing negative effects on employees is necessary.

Prior studies examined the effects of internal motivation and job characteristics on job satisfaction and job stress, autonomy, task identity, and task significance to improve job stress and feedback for increasing job satisfaction in the workplace [75]. Job characteristics effectively predict the job satisfaction and organizational commitment of employees [76]. However, to handle external environment change, Fu et al. [77] suggested that human source managers enhance education and training, job characteristics, and job adaptability to prompt organizational performance and find a competitive advantage in the contemporary industry. Grobelna [78] showed that job characteristics have a significant positive effect on employee job engagement, which, in turn, improves organizational outcomes. In sum, organizations need employees who are engaged with their work, and these employees usually exist as a result of proactivity, initiative, and responsibility for their work [79]. Furthermore, the tasks of employees in the modern-day industry are undoubtedly complex, with employees facing various challenges at work that may not be found in the standard operation procedure to handle any situation. Empowering leadership enhances employees’ autonomy and motivation at work by giving them feedback and opportunities and supports them in improving their skills and mastery of tasks [38]. According to Hackman and Lawler [69], the five core job characteristics (i.e., skill variety, task variety, task significance, autonomy, and feedback) are key elements for designing empowering work [80]. Moreover, skill variety, task significance, and autonomy are increased by task complexity [46]. Empowering leadership may thus be an important factor in encouraging employees to participate in decision making and exchanging the opinions necessary and essential to their organization [81]. Thus, we propose the following:

**Hypothesis** **2** **(H2).** *Empowering leadership has a positive influence on job characteristics.*

### 2.3. Relationship among Job Characteristics, Job Embeddedness, and Proactive Work Behavior

Job embeddedness is defined as the combined forces that keep employees from leaving their job and can be used to explain employees’ intention to leave [82]. Job embeddedness has three dimensions, namely, link, fit, and sacrifice, and this concept is needed in the present-day industry to understand employee turnover after controlling for job-related attitudes [53]. Based on conservation of resources theory, job embeddedness is developed as a result of the richness of individual resources in organizations [83], and job embeddedness has value in predicting important work outcomes and job performance [84]. Kiazad et al. [85] argued that the attitude of employees motivated to acquire and retain job resources explains their embedded behavior, and that organizations should find ways to increase employees’ instrumental social and psychological resources to advance their reactions to the loss of resources [86]. Felps et al. [87] demonstrated that colleagues’ job embeddedness and job search behavior have critical effects on explaining why people leave. Thus, job embeddedness is considered an effective solution for voluntary turnover [88]. Prior research found a negative relationship between organizational sacrifice and turnover intention, and the embeddedness concept appears to be a strong predictor of employees’ job performance and customer satisfaction [89]. Given that the high turnover rate of employees has been a particular concern in the contemporary industry, organizations should increase employees’ job embeddedness by improving their perceived turnover cost. Employees regard the support of colleagues and family as a work resource, which enhances their sense of belonging in their organization, and through job embeddedness performing creative performance [55]. Generating novel ideas to improve services and providing creative solutions to customer problems and requirements with colleagues are the expected goals of frontline service positions in the contemporary industry. Managers should create a work environment that encourages colleagues to support one another because effective teamwork can stimulate the group cohesiveness of employees in the workplace [90]. However, diversity atmosphere represents an important resource that employees wish to maintain by staying in their organization, thereby increasing the embeddedness in their organization and reducing their intention to leave [51].

Job characteristics theory is conceptualized as a person-environment fit [91], and job design characteristics determine employees’ work motivation, satisfaction, and outcomes [46,92]. That is, work design can determine the extent to which employees may be motivated to complete their tasks, have positive attitudes, and find meaning at work to stay in their organization [31]. Accordingly, employees likely stay in their job when it is enriched, and they feel that they fit well with their job [93]. In this vein, job characteristics, such as significance and identity, are confirmed to foster employees’ job embeddedness with low turnover intention [94]. Furthermore, high-performance work practices manifest that training, empowerment, and rewards enhance frontline employees’ work engagement, and such practices, in turn, touch off on job outcomes and extra-role customer service [16]. Similarly, job embeddedness represents the collection of factors that keep employees at work [51]. Employees’ work engagement can cause them to devote themselves and be further embedded in their job; in other words, they are unlikely to express an intention to leave [15,95]. Thus, job embeddedness can be a critical factor in job attitude and can have a positive effect on work outcomes. In this vein, job embeddedness makes employees more proactive than before [96], and employees with organizational embeddedness have been shown to be proactive at work and have a strong influence on excellent service performance [97]. Most of all, drawing from the theory of planned behavior that links individual beliefs to behavior [98], subjective norm, attitude, and perceived behavioral control are three core ingredients, which collectively develop an individual planned behavior. Following this perspective, job embeddedness can be considered a key indicator of behavioral attitude, employees’ willingness, and motivation to exhibit proactive work behavior. Therefore, we propose the following:

**Hypothesis** **3** **(H3).** *Job characteristics have a positive influence on job embeddedness.*

**Hypothesis** **4** **(H4).** *Job embeddedness has a positive influence on employees’ proactive work behavior.*

### 2.4. Serial Mediation Roles of Job Characteristics and Job Embeddedness

The above mentioned relationship refers to empowering leadership, job characteristics, and job embeddedness, which are related and have positive effects on proactive work behavior. Based on job demands-recourses and conservation of resources theories, job characteristics and job embeddedness are regarded as motivation processes in the psychology of employees and thus have effects on proactive work behavior and job performance [27,31]. In addition, resources are defined as anything based on individuals’ perceptions that can meet their goals [30]. The five core job characteristics are considered personal resources. According to job demands-recourses theory [26], job characteristics generate resources to prevent negative effects leading to positive performances [46]. Empowering leadership thus motivates engaged employees to embed in their organization [40]. That is, empowered employees can have utilized job resources to defy job demands and make changes in their work to display proactive work behavior [27]. Furthermore, job demands-recourses and conservation of resources theories have linked resources to employee job embeddedness [99]. Accordingly, a serial mediation process of job characteristics and job embeddedness for linking empowering leadership and proactive work behavior may exist, and empowering leadership can enrich jobs and enhance job design characteristics. Meanwhile, job characteristics can also significantly influence the job embeddedness of employees that can make them further proactive in their job. Employees may be conscious of job characteristics and increase their job embeddedness through empowering leadership and exert high levels of proactive work behavior. Hayes [100] suggested that a serial mediation model is essential for further understanding the causal linkages in an integrated model. Thus, the serial mediation model is proposed in this study to investigate the serial mediating effects of job characteristics and job embeddedness between empowering leadership and employees’ proactive work behavior. Accordingly, we propose the following:

**Hypothesis** **5** **(H5).** *Job characteristics mediate the effect of empowering leadership on employees’ proactive work behavior.*

**Hypothesis** **6** **(H6).** *Job embeddedness mediates the effect of empowering leadership on employees’ proactive work behavior.*

**Hypothesis** **7** **(H7).** *Job characteristics and job embeddedness jointly mediate the effect of empowering leadership on employees’ proactive work behavior.*

## 3. Methodology

### 3.1. Research Framework

**Figure 1:**
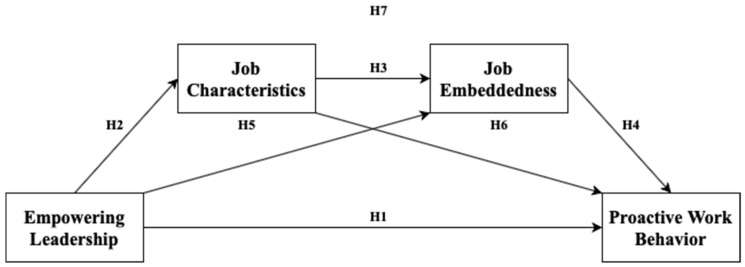
Research framework.

A serial mediation model with two mediators was developed to examine the hypotheses, with job characteristics and job embeddedness as the first and second serial mediators, respectively. Empowering leadership can influence the two serial mediators in order, leading to proactive work behavior. The research framework was proposed to investigate whether job characteristics and job embeddedness mediate the effect of empowering leadership on proactive work behavior with the serial mediation model (Figure 1).

### 3.2. Pilot Test

A pilot test was conducted to increase content validity and ensure the accuracy of the instruments in this study. The verification of reliability and validity of the measurement items were also evaluated before the formal data collection [101]. Pilot test data were collected from tourist hotel employees in southern Taiwan through stratified random sampling. Nunnally [102] suggested that the alpha coefficient should reach 0.70 for a satisfactory standard. A total of 109 hotel employees of 15–20 distinct departments and firms participated in the pilot phase, and the result showed high coefficients (all measurement items over 0.7 threshold) for each construct. Therefore, no items were deleted, and each item of construct was adequate for representing the validity.

### 3.3. Sample Frame and Data Collection

The focus of this study was on tourist hotels in Taiwan. According to the statistics data by the Taiwan Tourism Bureau in 2020, 124 tourist hotels are listed [103]. Our participants were employees of three- to five-star tourist hotels in northern (25.6%), central (14.1%), southern (48.8%), and eastern (11.5%) Taiwan. The data distribution is similar to the tourist hotel population structure in Taiwan. To avoid potential common method variance bias, stratified random sampling was utilized from each level containing five to 10 respondents of 30–60 distinct departments and organizations. Human resource managers were also contacted to ensure that they were willing to assist in this research. Nunnally [102] suggested that the sample size should be more than 10 times the number of items. Consequently, this study had 55 items, that is, the minimum sample size was 550. A total number of 550 questionnaires were collected, and 461 were valid samples representing an effective response rate of 83.8% and were utilized for analyzing data. The respondents were composed of 12 firms. Each firm had five to seven departments, and each department had 8 to 13 employees.

### 3.4. Construct Measurement

Each instrument has been widely used to assess the constructs being investigated. A seven-point Likert scale was adopted with scores ranging from 1 (strongly disagree) to 7 (strongly agree). Empowering leadership was measured using the 12-item scale of Leadership Empowerment Behavior [32], which corresponded to four dimensions: (1) enhancing work meaningfulness, (2) fostering participation in decision making, (3) expressing confidence in high performance, and (4) providing autonomy from bureaucratic constraints. The Cronbach’s alpha for this scale was 0.93; sample item: “My manager helps me understand how my objectives and goals relate to those of the company.” Next, 23 items were adapted from the Job Diagnostic Survey developed by Hackman and Oldham [70] to measure job characteristics. The scale items included five core dimensions, such as (1) skill variety, (2) task identity, (3) task significance, (4) autonomy, and (5) feedback. The Cronbach’s alpha of this scale was 0.82; sample item: “I have a chance to do a number of different tasks using a wide variety of skills and talents.” Then, job embeddedness was measured using a seven-item scale from a global measure from Crossley, et al. [104], with a Cronbach’s alpha of 0.89; sample item: “I feel attached to this organization.” Last, proactive work behavior was measured using a 13-item scale from Parker and Collins [66], as manifested in four dimensions: (1) taking charge, (2) individual innovation, (3) problem prevention, and (4) voice. The Cronbach’s alpha for this scale was 0.91; sample item: “How frequently do you attempt to bring about improved procedures in your workplace?” Demographic information, such as gender, age, work experience, education level, position, and job type of the participants were included as control variables.

### 3.5. Analytic Approach

Data screening and preparation were assessed using SPSS 24.0 and AMOS 24.0. Demographic profiles were demonstrated to identify respondents’ information. Structural equation modeling was also conducted to examine all hypotheses in this study with maximum-likelihood estimation [105]. On the basis of a two-step approach recommended by Anderson and Gerbing [106], confirmatory factor analysis (CFA) was conducted to assess construct validity. In CFA, common method factor, goodness of model fit test, convergent validity test, and discriminant validity test were considered before path analysis. For the goodness of model fit [107], preliminary fit criteria, fit of the internal structure of the model, and overall model fit criteria were evaluated using the overall chi-square (*χ*^2^), the degrees of freedom for the chi-square (*χ*^2^/*df*), the goodness of fit index (GFI), adjusted goodness of fit index (AGFI), standardized root mean square residual (SRMR), comparative fit index (CFI), non-normed fit index (NNFI), and root mean square error of approximation (RMSEA). Path analysis was performed to determine whether the data were consistent with the model and to confirm the hypotheses of this study. The mediating effects were confirmed using the bootstrap approach based on 10,000 bootstrapping samples with a 99% confidence interval (CI) [108].

## 4. Results

### 4.1. Demographic Statistics

Among the respondents (*n* = 461), demographic data regarding gender, age, work experience, education level, position, and job type are presented in Table 1.

### 4.2. Confirmatory Factor Analyses

Confirmatory factor analysis is a multivariate statistical procedure to examine how well the measured items represent the nature of constructs in a hypothesized measurement model. Table 2 shows the four constructs of empowering leadership, job characteristics, job embeddedness, and proactive work behavior, which were evaluated by the degrees of freedom for the chi-square (*χ*^2^/*df*) and had good scoring range from 1.52 to 2.11. As for the domain of absolute fit indices, the constructs reached satisfactory results with the goodness of fit index (GFI) score ranging from 0.93 to 0.98, receiving satisfactory results with the adjusted goodness of fit index (AGFI) score range from 0.93 to 0.97 and indicated satisfactory results with the standardized root mean square residual (SRMR) score lower than 0.08, except for job characteristics with SRMR score = 0.16. In terms of incremental index, constructs achieved satisfactory results with the comparative fit index (CFI) score around 0.98 to 0.99, non-normed fit index (NNFI) also indicated convincing consequences with the scores around 0.97 to 0.99. The root mean square error of approximation (RMSEA) of all constructs satisfied the recommended score around 0.03 to 0.05. Thus, the proposed model of this study had better outcomes and passed the goodness of model fit.

Table 3 shows the means, standard deviations, and correlation coefficients. The analysis provides support for considering a method for conducting the investigation. The preliminary results indicate that the construct reliability of each construct ranged from 0.80 to 0.88, exceeding the 0.60 threshold values [109] which is evidence for the convergent validity of the model. Meanwhile, the average variance extracted also indicates the results with the score range from 0.41 to 0.65, exceeding the 0.36 acceptable threshold [109]. On the other hand, the square root of average variance extracted among each construct must be higher than the correlation, and the total number of qualified correlation values of each construct must at least account for 75% [110]. Accordingly, the total estimated correlations of variables resulting in good discriminant validity is lower than the square root of average variance extracted in each construct. Goodness-of-fit test, convergent validity test, and discriminant validity test were all verified and qualified through confirmatory factor analysis. The proposed model demonstrated good significance and suitability for path analysis.

Harman’s single factor analysis was conducted to reduce the concern of common method variance bias, the principal factor explained 33.45% of the variance, not exceeding 50% threshold [111]. Table 4 shows the chi-square test by Gaskin [112]. The analysis mitigates the common method bias with comparison of the unconstrained and the fully constrained common method factor model. A chi-square difference test indicates a significant *p*-value (*p* = 0.000). Therefore, common method variance bias is not a serious problem in the dataset.

### 4.3. Path Analysis

**Figure 2:**
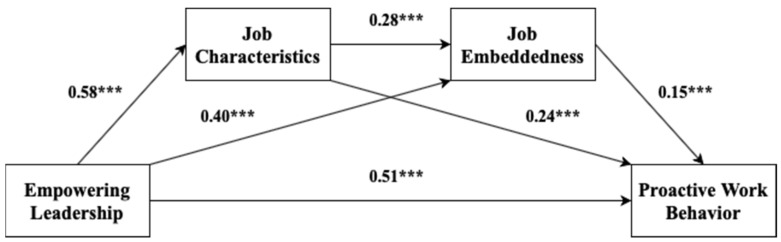
Path analysis of this study Note. *** *p* < 0.001.

Figure 2 shows that empowering leadership had a significant direct effect on proactive work behavior (path coefficient = 0.51, *p* < 0.001) and a positive and significant effect between empowering leadership and job characteristics (path coefficient = 0.58, *p* < 0.001). Job characteristics also had a significant direct effect on job embeddedness (path coefficient = 0.28, *p* < 0.001) and job embeddedness had a significant direct effect on proactive work behavior (path coefficient = 0.15, *p* < 0.001). Thus, Hypothesis 1 to Hypothesis 4 were confirmed (see Table 5).

Furthermore, the analysis investigated the indirect effect of empowering leadership on employee proactive work behavior via job characteristics and job embeddedness and percentile bootstrapping. Bias-corrected percentile bootstrapping was signified with 10,000 bootstrap samples at a confidence interval level of 99% following Taylor et al. [108]. The first indirect path of empowering leadership affects proactive work behavior and job characteristics had a mediating role in the relationship between empowering leadership and proactive work behavior (*β* = 0.14, *p* < 0.001). The lower and upper bounds of the 99% CI did not comprise 0 [lower bound CI = 0.09, upper bound CI = 0.20]. Therefore, Hypothesis 5 was supported. The results also demonstrated that the effect of empowering leadership on proactive work behavior was also mediated by job embeddedness (*β* = 0.06, *p* < 0.001). The lower and upper bounds of the 99 percent CI did not include 0 [lower bound CI = 0.02, upper bound CI = 0.11], supporting Hypothesis 6. Eventually, the results from the serial mediation analysis indicated a serial mediation model of job characteristics and job embeddedness in the relationship between empowering leadership and proactive work behavior. Linking empowering leadership and proactive work behavior through job characteristics and job embeddedness was significant (*β* = 0.02, *p* < 0.001). The lower and upper bounds of the 99 percent CI did not involve 0 [lower bound CI = 0.01, upper bound CI = 0.05]. The interpretation is that job characteristics and job embeddedness are variables that explain why employees tend to perform a higher level of proactive work behavior. Consequently, Hypothesis 7 was supported (see Table 5).

## 5. Discussion

### 5.1. Theoretical Implications

Based on job demands-recourses and conservation of resources theories, the study aims to investigate the effect of empowering leadership on proactive work behavior with a serial mediation model, which applies job characteristics and job embeddedness as mediators. A comprehensive integrated model is also proposed by combining both theories for explaining and predicting the motivation and attitude of employees. This study broadens previous research in threefold. First, although the relationship between empowering leadership and proactive work behavior has been examined [49], research that extends proactive behavior studies, which provide an evidenced integrated model, is lacking [44,45]. Our results therefore contribute to existing literature, which indicates that empowering leadership directly and indirectly influence proactive work behavior in a serial mediation way. Second, our results support that job characteristics and job embeddedness play significant roles in influencing proactive work behavior through empowering leadership. This study is one of the leading investigations that attempts to consider two mediators in an integrated model in the contemporary industry. Last, prior studies regarded job characteristics and job embeddedness as critical factors in organizational performance management [54,55]. However, only few studies can provide comprehensive investigations of the effects on proactive work behavior through empowering leadership. Accordingly, our study provides a new perspective that job characteristics and job embeddedness can be regarded as powerful resources to escalate the competitiveness level in sustainable human resource management practices for workplace mental health.

Moreover, high turnover rates in the contemporary industry have long been the focus of research. This study develops a sustainable human resource management and provides valid grounds for industry practitioners. Drawing from high-performance work practices, an empowering strategy is adopted to pull employees to be proactive in their job and to leverage the benefits in their organization. Empowering leadership can enrich the job design characteristics, which make employees stay in their job and have the willingness to stay in their organization for the long term [30,31]. Employees are highly reliant on interactions with customers, and organizational embeddedness makes them further proactive at work [96], which fosters excellent job performance and workplace mental health [97]. That is, job characteristics and job embeddedness are critical motivational mechanisms in the indirect positive effects of empowering leadership and proactive work behavior.

Scholars have argued about an urgent requirement to find strategies to support people and promote positive mental health in the workplace [1,2,3]. This study advances scholarly understanding on why empowering leadership influences employee proactive work behavior by integrating job demands-recourses and conservation of resources theories. The findings suggest that empowering leadership is associated with proactive work behavior through a serial mediation process with two mediators, namely, job characteristics and job embeddedness. By addressing the importance of workplace mental health, organizations can engage employers in good practice for overcoming risks and improving employees’ productivity. Therefore, the ability of maintaining mental health has a beneficial effect on proactive behavior at work and can be regarded as a critical element of work-related performance [8]. Importantly, this study is the first attempt to investigate the joint effect of two mediations and broaden previous research. In the past, the effect of job characteristics on job embeddedness was investigated, and significant implications for turnover intention and positive outcomes were observed [57]. However, the relationship between empowering leadership and proactive work behavior has not been discussed. Given that the loss of resources is occurring fast, gaining additional resources is necessary [28]. Wrzesniewski and Dutton [113] argued that employees may proactively change to make their tasks more meaningful than before, enhance their relationships with clients and colleagues, improve the cognition of their work, and even increase their job sources and decrease their job demands [114]. Therefore, the results of our integrated model support the idea that employees tend to take a proactive approach to ask for feedback, learn a new skill or reduce bureaucracy, and optimize their workplace and working environment [27]. In this vein, empowering strategies reconcile bureaucratic constraints and enhance work meaningfulness, thereby enabling employees to work confidently and with high performance for workplace mental health [32].

### 5.2. Managerial Implications

Given that employees have become the most valuable asset for any organization, especially in the service industry, employee participation is regarded as one of the core characteristics of sustainable human resource management for workplace mental health. Accordingly, frontline employees are key workers who interact directly with customers; each action of frontline employees can bring customers the most intuitive and subjective feelings and is a crucial part of organizational assessment. Even though back-office employees may never meet a customer, they are nonetheless cogs in the wheel to performance management. An old saying is that a little leak will sink a great ship. As such, employee orientation and training are indispensable for all employees.

Managers are often overwhelmed with heavy workload and thus, for the consistent changes in the contemporary organizational structure, empowering leadership can reduce their workloads and give employees a free hand, so that they can discover and solve problems quickly. Empowering leadership also has a significant influence on employees’ attitude and behavior. Enriched jobs make employees further tied to their tasks through job embeddedness, which may assist organizations in achieving incremental organizational competitiveness and decrease employees’ high turnover intentions. The service-type business has a high uncertainty of service, and plenty of situations and customer problems require making quick decisions. Proactivity is the most necessary when it occurs [115]. Thus, employees should be trained to manage their job performance with high-performance work practices in a consistent way. Despite proactive employees being able to bring considerable advantages and work outcomes, proactive behavior at work is still not always effective and sometimes has negative consequences. For this reason, employees should consider when to be proactive, such as for tasks and strategies (i.e., whether proactivity is needed and what types of changes are needed in the situation), others’ views, supervisors’ reactions, and self-regulation [116].

Considering that sustainable human resource management has been used to decide the strength of a human resource management system and can create organizational value (e.g., change management, productivity, innovativeness, and profitability), societal value (e.g., firm image, community well-being, and employment growth), and employee value (e.g., motivation, good work-life balance, engagement, and employee well-being) [19,22], industry practitioners can use sustainable human resource management for offering a new approach to manage people, preserve productivity, and retain quality employees. Most of all, with a proper sustainable human resource management system, employee participation, open communication, and flexibility at work certainly play the salient roles of human health and job performance [23]. Therefore, sustainable human resource management enables the achievements of the organizations, societies, and employees of contemporary enterprises.

### 5.3. Research Limitations and Future Suggestions

Our findings must be interpreted with caution in light of some limitations. First, this study exclusively examined the positive outcomes of empowering leadership, but a comparison with other leadership styles was not conducted to explain what proactive work behavior can be attributed to a specific leadership style. Employees’ desired behavior is also necessary for contemporary organizations. Thus, further studies can jointly consider multiple leadership styles to ensure the uniqueness of antecedent variables or replace proactive work behavior with other firm and valued outcomes. Second, although stratified random sampling, Harman’s single-factor analysis, and Gaskin’s method were used to reduce the concern of common method variance bias, future studies are suggested to use managerial data to prevent self-reported problems. In addition, the analysis of the development of the relationship between empowering leadership and proactive work behavior was limited in the hotel industry. Further analyses of the real economic impacts of such changes in human resource management or comparisons with other service industries or situations in other countries are encouraged to help make more general conclusions. Finally, our research design is a cross-sectional experiment. Future studies are thus encouraged to adopt a longitudinal research design to support further experimentation.

## 6. Conclusions

Drawing upon job demands-recourses and conservation of resources theories, this study found that empowering leadership has positive influences on job characteristics and proactive work behavior, whereas job characteristics have a positive influence on job embeddedness. The result also demonstrated that job characteristics and job embeddedness were keys underlying the mechanism to connect the relationship between empowering leadership and proactive work behavior. Therefore, the findings support that sustainable human resource management, which promotes empowering leadership for proactive work behavior, can be regard as an essential resource for human health and organizational value at work.

## Figures and Tables

**Table 1:** Respondent profiles.

Sample Characteristics (*n* = 461)	Frequency (s)	Percentage (%)
Gender		
Female	278	60.3
Male	183	39.7
Age		
20 or below	19	4.1
21–30	337	73.1
31–40	48	10.4
41–50	29	6.3
51–60	24	5.2
61 or above	4	0.9
Work experience		
Below 1 year	71	15.4
1 year or more, less than 3 years	218	47.3
3 year or more, less than 5 years	62	13.4
5 year or more, less than 7 years	27	5.9
7 year or more, less than 9 years	20	4.3
9 years of above	63	13.7
Education level		
Junior high school or below	7	1.5
Senior high school	49	10.6
Bachelor’s degree	379	82.2
Postgraduate degree or above	26	5.6
Position		
Managerial	78	16.9
Non-managerial	383	83.1
Job type		
Full-time	421	91.3
Part-time	40	8.7

**Table 2:** Summary of the goodness of model fit test.

Constructs	*χ* ^2^	*χ*^2^/*df*	GFI	AGFI	SRMR	CFI	NNFI	RMSEA
Empowering Leadership	78.59	1.57	0.98	0.97	0.05	0.99	0.99	0.04
Job Characteristics	345.54	1.52	0.93	0.93	0.16	0.98	0.97	0.03
Job Embeddedness	29.57	2.11	0.97	0.96	0.05	0.99	0.98	0.05
Proactive Work Behavior	120.86	1.98	0.97	0.97	0.05	0.99	0.98	0.05
Overall Model	257.88	1.57	0.96	0.95	0.09	0.98	0.98	0.04

**Table 3:** Mean, standard deviation, and correlation coefficient.

	Mean	S.D.	1.	2.	3.	4.
1. Empowering Leadership	5.10	0.88	(0.81)			
2. Job Characteristics	4.84	0.60	0.56 **	(0.67)		
3. Job Embeddedness	4.46	0.80	0.56 **	0.52 **	(0.64)	
4. Proactive Work Behavior	5.30	0.80	0.73 **	0.64 **	0.56 **	(0.79)

Note: number in the brackets represents the square root of average variance extracted. ** *p* < 0.01.

**Table 4:** Common method bias test results.

	Chi-Square	*df*	*p*-Value	Invariant?
Overall Model				
Unconstrained	709.83	144		
Fully Constrained	946.80	150		
Number of Groups		2		
Difference	236.97	6	0.000	NO

**Table 5:** Evaluations of serial mediation.

Hypothesis	Path	Estimate	*p*-Value	Percentile 99% CI [Lower, Upper]	Results
*H1*	EL → PWB	0.51	*p* < 0.001	[0.40, 0.61]	Supported
*H2*	EL → JC	0.58	*p* < 0.001	[0.49, 0.65]	Supported
*H3*	JC → JE	0.28	*p* < 0.001	[0.22, 0.55]	Supported
*H4*	JE → PWB	0.15	*p* < 0.001	[0.04, 0.26]	Supported
*H5*	EL → JC → PWB	0.14	*p* < 0.001	[0.09, 0.20]	Supported
*H6*	EL → JE → PWB	0.06	*p* < 0.001	[0.02, 0.11]	Supported
*H7*	EL → JC → JE → PWB	0.02	*p* < 0.001	[0.01, 0.05]	Supported
Total Indirect Effect		0.23	*p* < 0.001	[0.15, 0.30]	
Total Effect		0.73	*p* < 0.001	[0.66, 0.79]	

EL = empowering leadership, JC = job characteristics, JE = job embeddedness, PWB = proactive work behavior.

## Data Availability

Data available on request due to privacy.
